# Quantifying the Efficiency and Equity Implications of Power Plant Air Pollution Control Strategies in the United States

**DOI:** 10.1289/ehp.9712

**Published:** 2007-01-22

**Authors:** Jonathan I. Levy, Andrew M. Wilson, Leonard M. Zwack

**Affiliations:** Department of Environmental Health, Exposure Epidemiology and Risk Program, and Harvard Center for Risk Analysis, Harvard School of Public Health, Boston, Massachusetts, USA

**Keywords:** environmental justice, equity, particulate matter, power plant, premature mortality, risk assessment

## Abstract

**Background:**

In deciding among competing approaches for emissions control, debates often hinge on the potential tradeoffs between efficiency and equity. However, previous health benefits analyses have not formally addressed both dimensions.

**Objectives:**

We modeled the public health benefits and the change in the spatial inequality of health risk for a number of hypothetical control scenarios for power plants in the United States to determine optimal control strategies.

**Methods:**

We simulated various ways by which emission reductions of sulfur dioxide (SO_2_), nitrogen oxides, and fine particulate matter (particulate matter < 2.5 μm in diameter; PM_2.5_) could be distributed to reach national emissions caps. We applied a source–receptor matrix to determine the PM_2.5_ concentration changes associated with each control scenario and estimated the mortality reductions. We estimated changes in the spatial inequality of health risk using the Atkinson index and other indicators, following previously derived axioms for measuring health risk inequality.

**Results:**

In our baseline model, benefits ranged from 17,000–21,000 fewer premature deaths per year across control scenarios. Scenarios with greater health benefits also tended to have greater reductions in the spatial inequality of health risk, as many sources with high health benefits per unit emissions of SO_2_ were in areas with high background PM_2.5_ concentrations. Sensitivity analyses indicated that conclusions were generally robust to the choice of indicator and other model specifications.

**Conclusions:**

Our analysis demonstrates an approach for formally quantifying both the magnitude and spatial distribution of health benefits of pollution control strategies, allowing for joint consideration of efficiency and equity.

In many settings there are tensions between efficiency and equity in deciding on optimal pollution control strategies. Within the context of benefit-cost analysis, efficiency may be related to implementing the least-cost control strategy to achieve a given health benefit, or alternatively, to maximizing net benefits. Similarly, equity can involve procedural fairness (i.e., equal involvement in public proceedings) or equity in the distribution of outcomes (Jacobson et al. 2005). Inequity consists of those inequalities that may be considered unjust or unfair (Macinko and Starfield 2002). Although there are multiple interpretations of these terms, we focus here on efficiency as maximizing the public health benefits of a control measure, and on equality in the distribution of those benefits across at-risk individuals as the dimension of equity that can be included in quantitative analysis.

Given these definitions, although efficiency is incorporated into any health benefits analysis, equity and related distributional issues are often omitted (Yitzhaki 2003). Most regulatory impact analyses have focused exclusively on aggregate benefits [U.S. Environmental Protection Agency (EPA) 1999a, 1999b] without formally considering the geographic or demographic distributions of these benefits. In parallel, many studies of equity or environmental justice did not quantify health risks, instead focusing on proximity to sources (Burke 1993; Pollack and Vittas 1995; Sheppard et al. 1999), emissions (Millimet and Slottje 2002a, 2002b; Perlin et al. 1995), or concentrations (Lopez 2002). Studies that quantified risk inequality (Apelberg et al. 2005; Morello-Frosch and Jesdale 2006) or proposed a framework to do so (Finkel 1990, 1997) focused on characterizing baseline distributions of risk rather than the benefits of control strategies, and the appropriate methodology may differ in this context. The lack of a systematic framework to simultaneously consider efficiency and equity in a decision context may imply that decisions are based largely on maximization of societal benefits without formal consideration of equity implications.

To address these limitations, we developed a framework by which risk inequality could be formally quantified within health benefits analysis (Levy et al. 2006). Briefly, we proposed that quantitative indicators of inequality, similar to those used to measure income inequality, could allow decision makers to construct an optimal efficiency–equality frontier and avoid policies that are dominated across both dimensions. Based on an axiomatic approach, we selected the Atkinson index (Atkinson 1970) as the most appropriate indicator for health benefits analysis, focusing on the change in this indicator under different control scenarios. Other indicators were considered useful for sensitivity analyses (the Gini coefficient, mean log deviation, and the Theil entropy index).

Quantitative measures of risk-based efficiency and equality may be useful in many contexts, including the evaluation of national-level policies to control emissions from power plants in the United States. In theory these policies could involve site-specific control requirements or cap-and-trade programs. Cap-and-trade programs are designed primarily for economic efficiency but operate under the presumption that health benefits would be similar regardless of the distribution of emissions (Farrell and Lave 2004). However, given differences in atmospheric conditions and population patterns, how emission controls are distributed geographically could influence the magnitude and distribution of benefits. Sulfur dioxide (SO_2_) emission trading related to the Title IV Acid Rain Program (U.S. EPA 2007) resulted in greater health benefits than a hypothetical program without trading, based on the geographic distribution of controls (Burtraw and Mansur 1999).

Regardless of efficiency claims, environmental justice advocates and communities housing power plants have expressed concern that unrestricted emission trading does not decrease and may exacerbate environmental inequities (Solomon and Lee 2000). Previous analyses (Corburn 2001; Swift 2001) focused on the possibility of emissions hot spots associated with Title IV and whether low-income or minority populations tended to have lesser emission reductions in proximate facilities. While these studies concluded that there were no hot spots, they used a procedural rather than an outcome-based concept of equity and therefore did not address the question of changing patterns of health risks. The benefits analysis of Title IV (Burtraw and Mansur 1999) indicated that certain geographic areas received health benefits while others had health disbenefits. However, without a more formal analysis, it is difficult to determine whether health inequality increased, decreased, or stayed the same, or to ascertain the potential impacts of future policies. Given the framing of the debate about national power plant controls, an outcome-based focus implies that an evaluation of how various distributions of emission controls correspond to changes in health benefits and in the spatial inequality of health risk would be informative for the design of future emission control programs.

In this analysis, we focus on the various ways by which emissions reductions for power plants in the United States could be distributed to meet hypothetical national emissions caps for SO_2_, nitrogen oxides (NO_x_), and primary fine particulate matter (particulate matter with a diameter < 2.5 μm; PM_2.5_). For each control scenario, we estimate both the public health benefits and the change in the spatial inequality of health risk. We consider the sensitivity of our conclusions to the pollutants evaluated, the inequality indicators selected, and other factors.

## Methods

### Control scenarios

Given our objective of evaluating potential efficiency–equality tradeoffs, we needed to construct a number of control scenarios that spanned the efficiency–equality space and were interpretable. First, we established a national target emissions cap for all three pollutants. The Clear Skies Initiative (U.S. EPA 2003a) called for United States power plant SO_2_ emissions of 3 million tons and NO_x_ emissions of 1.7 million tons by 2018. The Clean Air Interstate Rule (CAIR) (U.S. EPA 2005a) called for power plant SO_2_ emissions of 3.5 million tons and NO_x_ emissions of 2.2 million tons by 2015. Alternative proposals have suggested caps of 2.2 million tons of SO_2_ and 1.5 million tons of NO_x_ (U.S. EPA 2001). As power plant emissions in 1999 (the base year for our analysis) were 12.6 million tons of SO_2_ and 5.7 million tons of NO_x_ (U.S. EPA 2003b), we consider 75% reductions in each to be generally representative of proposed regulations. Although primary PM_2.5_ was not incorporated into these proposals, controlling these emissions is plausible, given available technology and fuel options, and we consider a 75% reduction for consistency (but present our findings both with and without primary PM_2.5_ emissions).

For our control scenarios, our objective is not to simulate economic conditions and resulting plant behaviors or to consider the impact of current or pending regulations but simply to consider ways in which an aggregate 75% reduction could theoretically be distributed. We constructed some specified control scenarios that either reflect straightforward control policies or would provide bounding estimates of efficiency or equality regardless of their viability (Table 1). For example, all plants could have 75% emission reductions (scenario A) or all plants could meet a target emission rate per unit heat input, with variable percentage reductions (scenario B).

Scenarios C through P (Table 1) represent bounding values rather than realistic control scenarios and may miss important combinations of emissions reductions. To develop other scenarios, we used a simulation approach. For each pollutant, we allowed each plant to potentially have no change, control to a target emission rate per unit heat input, control halfway between current emissions and the target rate, or control to half the target rate. Or, a plant could shut down, eliminating all emissions. We iterated randomly across these options for all plants, and in each iteration, retained the scenario if total emissions of each of the three pollutants were within 5% of the target national emissions cap. We constructed 20 of these intermediate control scenarios.

### Source–receptor matrix

To link these emission changes with changes in ambient concentrations, we apply a source–receptor (S-R) matrix that has been used in previous regulatory impact analyses (U.S. EPA 1997, 1999a). S-R matrix is a reduced-form model that provides the relationship between emissions of PM_2.5_ or particle precursors and county-level PM_2.5_ concentrations. It is based on the Climatological Regional Dispersion Model (CRDM), a sector-averaged Gaussian dispersion model that includes wet and dry deposition and first-order chemical conversion of SO_2_ and NO_x_ to sulfate and nitrate particles.

S-R matrix includes county-specific calibration factors to adjust initial model outputs to reflect ambient monitoring data. Data from the U.S. EPA Federal Reference Method and Speciation Network monitors were spatially interpolated to county centroids, and the ratios between these values and the initial model outputs were used to develop calibration factors (Abt Associates 2006). The calibration factors had a median value of 0.9, indicating that relatively little bias was found in initial S-R matrix outputs, although there was some spatial variability (5th percentile of 0.5, 95th percentile of 1.4, range of 0.11–3.5).

### Power plant characteristics

We estimated emissions from the Emissions and Generation Resource Integrated Database (EGRID; U.S. EPA 2005b) and the National Emission Inventory (NEI; U.S. EPA 2005c). EGRID contained information on annual NO_x_ and SO_2_ emissions as well as heat input and electricity generation. We used power plant characteristics from 1999 for comparability with other available data. NEI provided information on annual PM_2.5_ emissions. Power plants were omitted from our analysis if they had been deactivated before 1999, if emissions data were unavailable, or if concentration modeling had not been conducted for all three pollutants within S-R matrix. The resulting database included 425 power plants, with total emissions in 1999 of approximately 11.8 million tons of SO_2_, 5.0 million tons of NO_x_, and 600,000 tons of primary PM_2.5_, indicating that our model captures most national power plant emissions.

### Demographics and concentration–response functions

We focus on premature mortality, as it contributed a majority of PM_2.5_-related benefits in previous health impact analyses (U.S. EPA 1999b, 2004). We derive our concentration–response function from the American Cancer Society cohort study (Pope et al. 2002), as this study has been used for the primary estimates in other health impact analyses (U.S. EPA 1999b, 2004) and has the largest and most geographically diverse population of available cohort studies.

For all-cause mortality, Pope and colleagues reported that mortality rates increased by 6% (95% confidence interval, 2–11%) for a 10-μg/m^3^ increase in annual average PM_2.5_ concentrations (using average concentrations across the study period), for a population age 30 and older. We collected population data for each county from 2000 Census data (U.S. Census Bureau 2005) and gathered background mortality data for each county from the CDC WONDER database, provided by the Centers for Disease Control and Prevention (CDC 2005). To provide more stable estimates, all-cause mortality data were aggregated across the years 1990–1998.

### Inequality indicators

In this section, we briefly describe the Atkinson index and the additional indicators relevant for sensitivity analysis, with more detailed information available in Appendix A and elsewhere (Levy et al. 2006).

The quantitative expression for the Atkinson index is


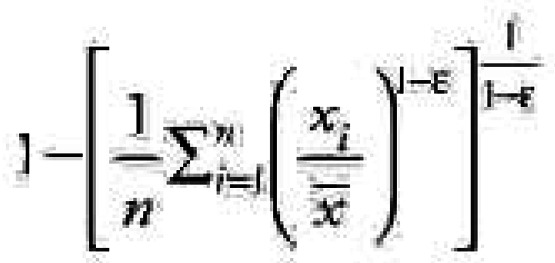


where *x_i_* represents the health risk for each individual, *n* represents the number of individuals affected, and ɛ is an explicit inequality parameter (ɛ of 0 implies no societal concern about inequality, with increasing values indicating greater aversion toward inequality). With the Atkinson index, the risk analyst need not decide *a priori* what the societal viewpoint about inequality should be, and can instead consider if policy decisions are sensitive to the value of ɛ.

The Atkinson index ranges from 0 to 1, with 0 representing complete equality and 1 representing maximum inequality. Because we are concerned about changes in inequality associated with control strategies, we focus on the difference between the Atkinson index of the precontrol distribution of concentrations or health risks and the Atkinson index given postcontrol concentrations or health risks.

In sensitivity analyses, we also consider the Gini index, the mean log deviation, and the Theil entropy index. The Gini index is defined as one-half the relative mean difference, or the average of the absolute differences between all pairs of values. The Gini index has a number of limitations in the context of health benefits analysis (Levy et al. 2006) but allows us to consider sensitivity to the approach for individual comparisons and aggregation. The mean log deviation is the average of the logarithm of the ratio between the mean health risk and the individual health risks *x_i_*. The Theil entropy index is similarly structured, averaging the product of the ratio between the individual health risks *x_i_* and the mean health risk and the logarithm of this ratio. Both these terms are in the same family of indicators as the Atkinson index and capture similar general concepts but without an explicit inequality aversion parameter and with other limitations (Levy et al. 2006). More detail about the calculation of the inequality indicators is provided in Appendix A.

### Sensitivity analyses

Although quantitative uncertainty analysis is beyond the scope of this analysis, we test the sensitivity of our conclusions to key model assumptions. For our base case, we quantify mortality benefits considering control of SO_2_, NO_x_, and PM_2.5_ jointly and using the Atkinson index to quantify spatial inequality. A decision must also be made about the relevant baseline against which to compare changes in mortality rates. Omission of baseline distributions of risk would lead to somewhat arbitrary determinations of inequality (Levy et al. 2006), but multiple baselines could be considered—all-cause mortality, PM_2.5_-related mortality, or power plant PM_2.5_-related mortality could be the outcome for which policymakers would hope to reduce inequality across the population with this hypothetical regulation. We consider PM_2.5_-related mortality in our base case.

For our sensitivity analyses, we consider pollutants separately and jointly, model tradeoffs for concentrations and health effects, and consider different definitions of baseline and different inequality indicators. We also present results calculating the inequality of the change in risk rather than the change in the inequality of risk: Atkinson (precontrol—postcontrol) rather than Atkinson (precontrol)–Atkinson (postcontrol). This approach effectively ignores baseline conditions and is not theoretically justified but helps us understand whether this erroneous approach leads to different conclusions. We present our primary results with ɛ = 0.75 for the Atkinson index, an illustrative value in the middle of the range typically found in the literature (Atkinson 1970; Kawachi and Kennedy 1997) but test values across a broad range.

## Results

**Figure 1:**
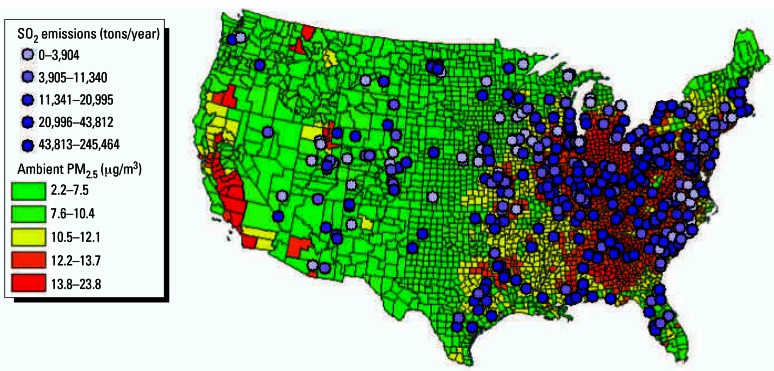
Annual average PM_2.5_ concentrations and SO_2_ emissions from 425 power plants in the United States.

Figure 1 presents the spatial patterns of annual average PM_2.5_ concentrations across the United States as well as the SO_2_ emission rates of the power plants in our analysis. Most of the high-emitting power plants are in the eastern United States, where PM_2.5_ concentrations are generally elevated.

**Figure 2:**
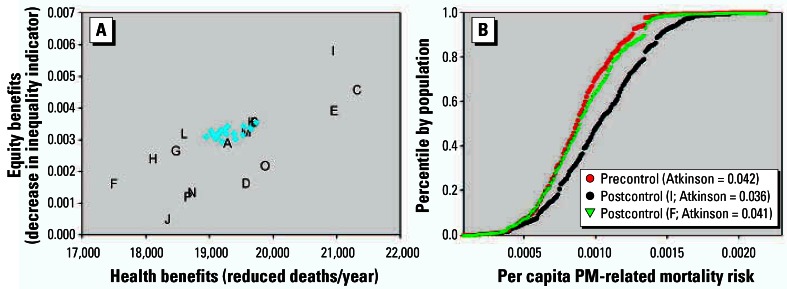
Annual mortality benefits and change in risk inequality for power plant control scenarios (*A*), along with distribution of risk for baseline conditions and selected control scenarios (*B*) (indicator = Atkinson index, ɛ = 0.75; pollutants = SO_2_, NO_2_, PM_2.5_; baseline = PM-related mortality). Blue dots in *A* represent intermediate control scenarios, and letters represent defined scenarios listed in Table 1.

We first consider the scenarios to simultaneously control SO_2_, NO_x_, and primary PM_2.5_, and apply the Atkinson index with ɛ = 0.75, calculating inequality based on changes in mortality risk from a baseline of PM_2.5_-related mortality. As indicated in Figure 2, the estimated public health benefits of the policies range from approximately 17,000 to 21,000 fewer premature deaths per year across control scenarios. Of this total, approximately 14,000–17,000 are associated with secondary sulfate particles. Given this, it is not surprising that the scenario with the greatest benefits (scenario C) involves controlling the plants with the highest health benefits per ton of SO_2_ emissions first. Policies requiring uniform emission reductions or for each plant to reach a target emission rate tend to fall in the middle of the efficiency spectrum, similar to the intermediate control scenarios.

**Figure 3:**
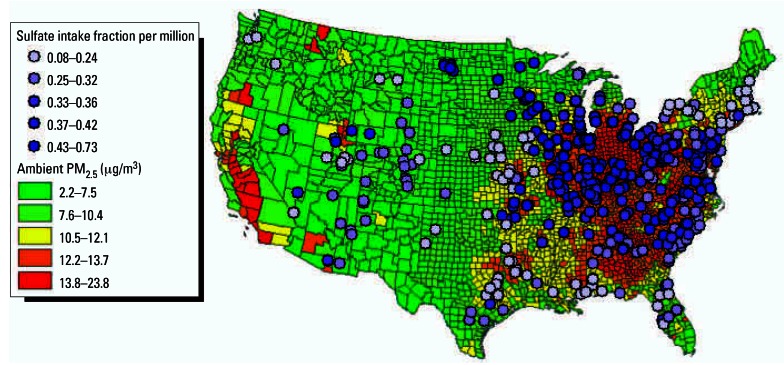
Power plant sulfate intake fractions and ambient PM_2.5_ concentrations. Intake fraction is a unit-less measure representing the sulfate population exposure per unit emissions of SO_2_, normalized by a nominal population breathing rate.

As the *y*-axis in Figure 2 represents the Atkinson index for postcontrol conditions subtracted from the Atkinson index for precontrol conditions, positive values indicate reductions in inequality and points toward the upper right represent more efficient and more equitable outcomes. Scenarios with greater health benefits generally also most reduce spatial inequality (Figure 2). This is because the power plants with maximum population exposure reductions per unit emissions of SO_2_ tend to be in the areas with highest ambient PM_2.5_ concentrations (Figure 3). The two policies on the optimal frontier involve controlling the plants with the highest health benefits per ton of SO_2_ emissions first (scenario C) or controlling the plants with the highest background PM_2.5_ concentration first (scenario I)—whether one prefers one policy over another depends on one’s willingness to trade efficiency for equality. All other policies are strictly dominated.

**Figure 4:**
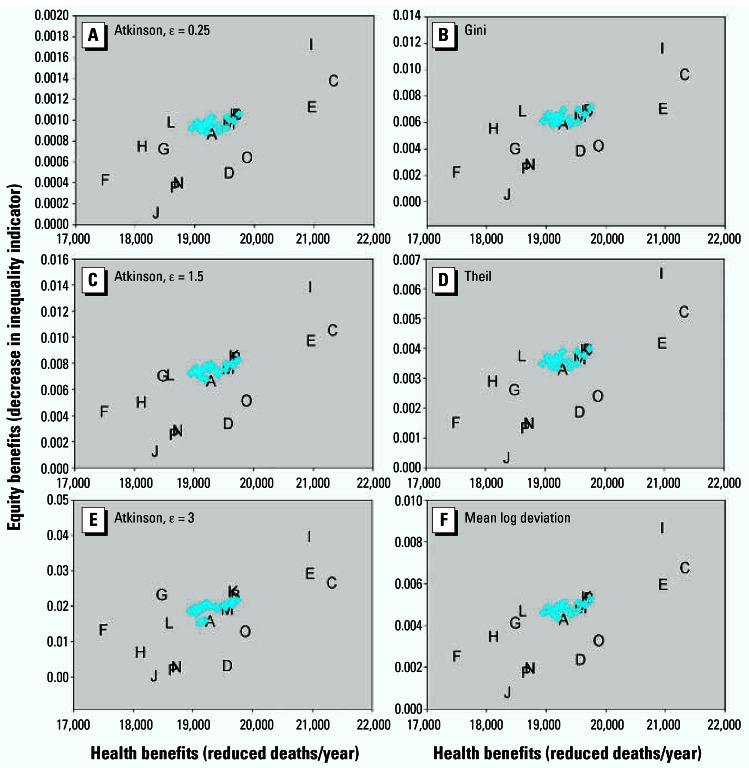
Sensitivity of efficiency–equality tradeoff conclusions to choice of inequality indicator, with model otherwise specified as in Figure 2. Inequality indicators: (*A*) Atkinson index, ɛ = 0.25; (*B*) Gini coefficient; (*C*) Atkinson, ɛ = 1.5; (*D*) Theil entropy index; (*E*) Atkinson, ɛ = 3; (*F*) mean log deviation.

Within our sensitivity analyses, we first considered the application of the Atkinson index with different values of ɛ as well as the other inequality indicators, holding other assumptions as in Figure 2. Of note, comparing the absolute values of the different inequality indicators to one another is not directly interpretable; the key question is whether the optimal policies are robust to the choice of indicator. Although there was some modest reordering of control strategies, the general conclusions remained robust, with only scenarios C and I on the optimal frontier (Figure 4). We present results for ɛ ranging from 0.25 to 3 (the range generally used in the literature), but conclusions are similar for higher values as well.

**Figure 5:**
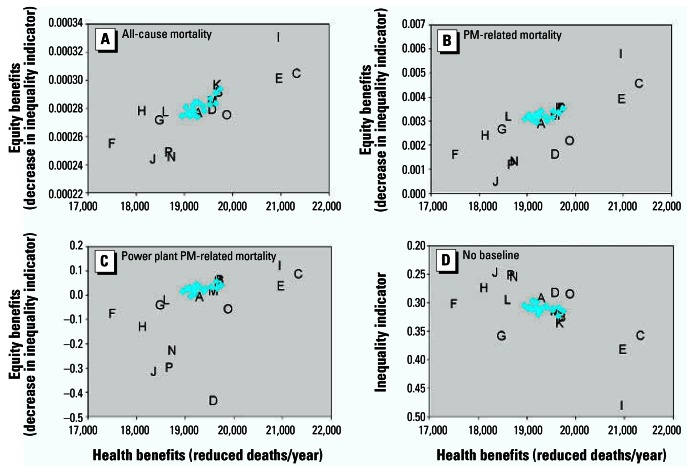
Sensitivity of efficiency–equality tradeoff conclusions to choice of baseline, with model otherwise specified as in Figure 2. Baselines: (*A*) All-cause mortality; (*B*) PM-related mortality; (*C*) power plant PM-related mortality; and (*D*) no baseline. The *y*-axis in *D* represents the inequality indicator itself rather than a change in the inequality indicator, and the axis is inverted so that more equitable scenarios remain in the upper-right quadrant of the graph.

Considering the influence of the choice of baseline highlights some important issues (Figure 5). Although the optimal strategies are identical for different mortality-related baselines, the Atkinson index changes to a greater extent for power plant PM_2.5_-related mortality. Postcontrol power plant-related PM_2.5_ mortality is close to zero in some locations, and the Atkinson index and other indicators are sensitive to near-zero values. Of greater significance is the fact that ignoring baseline conditions leads to substantially different conclusions, in which the scenarios previously considered to most improve equality are now considered to be least equitable. This is because scenarios such as C and I focus controls in geographic areas that have elevated baseline exposures and risks, so that the benefits are spread less uniformly but serve to reduce existing inequalities.

**Figure 6:**
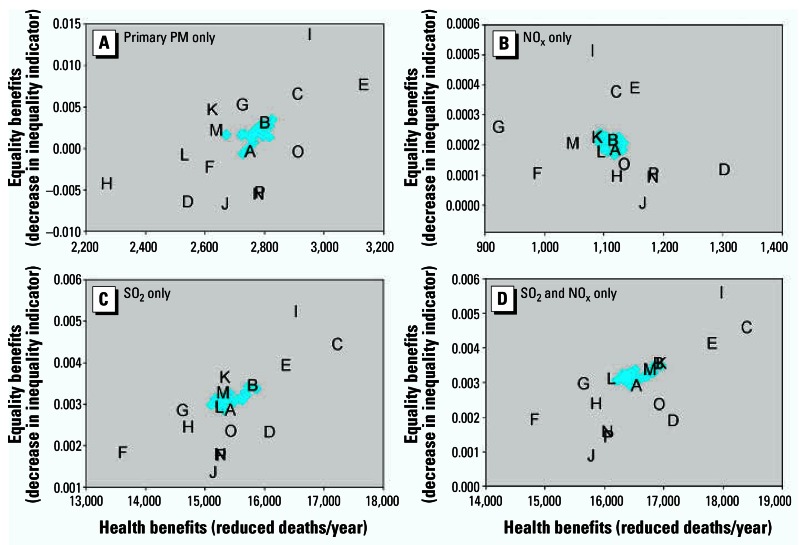
Sensitivity of efficiency–equality tradeoff conclusions to pollutants included in the model, with model otherwise specified as in Figure 2. Pollutants included in the model: (*A*) primary PM only; (*B*) NO_x_ only; (*C*) SO_2_ only; and (*D*) SO_2_ and NO_x_ only.

We additionally examined whether the conclusions differed when considering concentrations rather than health effects (with population-weighted concentration change as the efficiency measure and inequality in concentrations as the equity measure), with no significant difference in the findings (results not shown). Omission of primary PM_2.5_ emissions from the analysis, which more closely mirrors some of the proposed national cap-and-trade programs, led to similar conclusions (Figure 6). Not surprisingly, the optimal policies differed if only single pollutants were considered (i.e., controlling only NO_x_ emissions), but the findings similarly illustrated bounding estimates for efficiency by controlling the maximum/minimum health benefits per unit emissions and bounding estimates for equality by controlling the power plants in high/low ambient PM_2.5_ settings first.

Finally, if we allow multiple parameters to vary simultaneously, our conclusions are largely unaffected. For example, under all combinations of inequality indicators, choice of baseline, and use of concentrations or health risks (controlling all three pollutants), scenario I remains the most equitable, whereas scenario C remains the most efficient.

## Discussion and Conclusion

Our analysis demonstrates good concordance between national power plant emission reduction patterns that maximize health benefits and those that best reduce spatial inequality in the distribution of air pollution-related risks. This concordance will not always exist. It is clear that reducing risks for the highest-risk individual first would both maximize efficiency and minimize inequality, presuming no differences in the costs or feasibility of controls. However, pollution control strategies are targeted at sources rather than at individuals. In this context, tradeoffs are likely, as the factors that influence efficiency differ from the factors that influence equality. Our finding is based on the spatial coincidence between population risk reductions (largely a function of downwind population density at long distance) and individual risk reductions (largely a function of high ambient PM_2.5_ concentrations close to the power plant). As shown in Figure 6, this coincidence is stronger for some pollutants (SO_2_) than for others (NO_x_).

We also demonstrated within our analysis that these conclusions were robust across numerous model configurations as long as baseline conditions are appropriately incorporated. In particular, the optimal policy choices did not vary with ɛ; if the conclusions were sensitive to ɛ, follow-up studies would be needed to determine the values that best capture priorities of stakeholders and decision makers.

Another interesting finding is that the difference in health benefits across the control scenarios is small in relative terms, with only a 22% difference between the minimum and maximum benefits. This can be attributed to the fact that the emission reductions are substantial enough to require controls at many facilities, reducing the variation between scenarios in spite of larger variations in plant-specific benefits (Figure 3). That being said, a 22% difference does reflect an absolute difference of nearly 4,000 deaths per year, which could be significant in determining optimal policies. In addition, if not all power plants were controlled at the same time, the differences between the scenarios would increase if discount rates were applied to benefits in future years.

Although our findings are generally robust, a number of limitations are important to recognize. First, we have only addressed one dimension of equity, by focusing on spatial variability in county-level mortality risks with a national focus. More conventionally, equity considerations in an environmental justice context consider racial and ethnic disparities, which are omitted from this analysis. Inclusion of effect modifiers such as educational attainment (Pope et al. 2002) or evaluation of morbidity outcomes with known demographic patterning could significantly influence spatial patterns of risk (Levy et al. 2002) and any conclusions about equity, especially if methods are used to decompose inequality between and within different subpopulations (Levy et al. 2006). Although these factors are clearly important, much of the outcome-based debate related to national power plant control strategies has revolved around spatial equity. In general, the equity measure utilized should be the one most informative to the decision-maker within the context of the policy question. In applications in which other dimensions of equity are central, particularly those involving mobile sources (where the spatial extent of impact is lesser and local socioeconomic and demographic factors may be more influential), other measures should be used.

In addition, for our results to be useful for decision making, the costs of control need to be included, with realistic control strategies rather than bounding values and randomly-generated scenarios. With plant-specific control costs, we could compare net monetized benefits with changes in the spatial inequality of risk (noting that cost information cannot be used directly in our inequality indicator). Considering other dimensions of equity, such as the distribution of costs across power plant companies or consumers, would lead to a more comprehensive and relevant analysis, and methods should be developed to synthesize these elements into a single decision framework.

Our findings are also dependent on the validity of S-R matrix. Although S-R matrix is simplified relative to state-of-the-science dispersion models, it has yielded similar health impact estimates as more advanced models (Abt Associates et al. 2000; Levy et al. 2003). It also has the benefit of explicit calibration with ambient monitoring data. Moreover, given the numerous sources and control scenarios in our analysis, a more intensive model would have been infeasible. We can corroborate our modeling to a limited extent by comparison with similar analyses of the benefits of national cap-and-trade programs. For example, the U.S. EPA analysis of CAIR used CMAQ to estimate benefits of 17,000 fewer premature deaths per year, with population-weighted PM_2.5_ concentration reductions of about 1.2 μg/m^3^ (U.S. EPA 2005d). Our corresponding estimates (for SO_2_ and NO_x_ control only) of 15,000–18,000 fewer premature deaths and 1.3–1.6 μg/m^3^, for a slightly more stringent emissions cap, compare favorably with these estimates, although this validates our efficiency measures to a greater degree than our equity measures (which rely on spatial concentration patterns). S-R matrix also does not include the influence of NO_x_ emissions on ozone formation. Including ozone-related health benefits could theoretically influence our findings, although previous studies have shown that PM_2.5_ dominates monetized benefits (U.S. EPA 2005d).

In addition, although we conducted multiple sensitivity analyses, some alternative assumptions could have significantly influenced our findings. In particular, if definitive information were available about the relative toxicity of different particle constituents, our conclusions could differ. That being said, Figure 6 demonstrates that control scenario I is on the optimal frontier for all pollutants and therefore would be robust across different toxicity assumptions. Alternative assumptions about concentration–response function nonlinearities or regional differences in concentration–response functions could also have important effects. In particular, thresholds in the concentration–response function would reduce the benefits outside the Midwest, potentially enhancing the differences between control scenarios but likely not changing the optimal control scenarios. Nonlinearities and large variations in baseline risks would also lead to greater differences between concentration-based conclusions and risk-based conclusions, thereby enhancing the importance of risk-based indicators. As the epidemiologic evidence did not provide strong support for any of these factors, we did not formally incorporate them into sensitivity analyses, but they could be considered in future analyses as the evidence base evolves. Of note, other uncertain parameters (like the magnitude of the concentration–response relationship) would not influence the core conclusions about optimal control strategies. More generally, formal uncertainty analysis related to both efficiency and equity measures would be required for any future decision making in this setting.

A final concern is related to the inequality indicators themselves. Although the indicators we used agree with an axiomatic approach proposed previously (Levy et al. 2006), there are some limitations. The parameter ɛ in the Atkinson index most influences sensitivity to low values, but in a health risk inequality context, we are more concerned about high values, so this represents an indirect mechanism for expressing concern about different segments of the risk distribution. We also observed the sensitivity of all indicators to values near zero, which can be influential given certain definitions of baseline. Because of these issues, development of novel inequality indicators specific to health benefits analysis may be warranted, although our conclusions were not sensitive to the statistical formulation of the inequality indicator.

More generally, although our framework helped to identify policies on the optimal frontier and policies that were strictly dominated, it is difficult to know to what extent decision-makers should be willing to trade off a given increase in health benefits for a given decrease in an inequality indicator. Further research is needed into the interpretation of small changes in inequality.

Limitations aside, our analysis provides some useful insights. First, our scenarios provide both bounding values and an indication of the types of targeted control strategies that would be most beneficial. For example, if the initial allocation of permits in a cap-and-trade program were weighted to encourage greater emission reductions in zones with high concentrations or high health benefits per unit emissions, it would increase the likelihood of both maximizing health benefits and minimizing spatial inequality in PM-related risk. More generally, our analysis provides insight about the power plants that are the best candidates for controls from a health benefits and health equality perspective. Coupled with control cost information, these insights could be used to design an optimal control regimen. Finally, from a methodologic perspective, we have demonstrated the viability of developing efficiency–equality tradeoff frontiers in the context of health benefits analysis. These tools can be applied retrospectively (i.e., to Title IV) or prospectively to determine optimal policy options, taking into account both efficiency and equality.

## Figures and Tables

**Table 1:** Specified control scenarios for power plant simulation.

Scenario	Definition
A	75% reductions in SO_2_, NO_x_, and primary PM_2.5_ from all plants
B	Reductions in SO_2_, NO_x_, and primary PM_2.5_ from all plants to meet the average target emissions in pounds per million Btu, with plants currently below the target constrained to no emissions increases
C	Elimination of plants until all caps are met, starting from the highest health benefit per unit emissions of SO_2_, going down
D	Elimination of plants until all caps are met, starting from the highest health benefit per unit emissions of nitrogen dioxide (NO_2_), going down
E	Elimination of plants until all caps are met, starting from the highest health benefit per unit emissions of PM_2.5_, going down
F	Elimination of plants until all caps are met, starting from the lowest health benefit per unit emissions of SO_2_, going up
G	Elimination of plants until all caps are met, starting from the lowest health benefit per unit emissions of NO_2_, going up
H	Elimination of plants until all caps are met, starting from the lowest health benefit per unit emissions of PM_2.5_, going up
I	Elimination of plants until all caps are met, starting from the highest background PM_2.5_ concentration, going down
J	Elimination of plants until all caps are met, starting from the lowest background PM_2.5_ concentration, going up
K	Elimination of plants until all caps are met, starting from the highest SO_2_ emitters, going down
L	Elimination of plants until all caps are met, starting from the highest NO_2_ emitters, going down
M	Elimination of plants until all caps are met, starting from the highest PM_2.5_ emitters, going down
N	Elimination of plants until all caps are met, starting from the lowest SO_2_ emitters, going up
O	Elimination of plants until all caps are met, starting from the lowest NO_2_ emitters, going up
P	Elimination of plants until all caps are met, starting from the lowest PM_2.5_ emitters, going up

## Appendix A

As indicated in the text, we use four inequality indicators within our analysis. We apply the Atkinson index for our primary analysis, and we use the Gini coefficient, the mean log deviation, and the Theil entropy index for sensitivity analyses. In each case we apply the indicator to the precontrol distribution of risks, then to the postcontrol distribution of risks, with the difference between these values used to construct the efficiency–equality frontiers. Within this appendix, we provide an illustrative calculation for each of the indicators (including multiple values of ɛ for the Atkinson index).

The formulas for the four inequality indicators are listed below:

Atkinson index

where ɛ = inequality aversion (range from 0 to infinity),

Gini index

Mean log deviation

and

Theil entropy index

Now suppose there were 10 geographic regions affected by a control strategy, with the baseline and postcontrol distributions of risk as presented in Table A1. Note that the baseline risks roughly correspond to the deciles of PM_2.5_-related mortality risks in our analyses, and the three control scenarios are meant to illustrate the implications of controls focused on the bottom, middle, and top of the distribution (with approximate 10% risk reductions). For simplicity, we presume equal numbers of people in each risk bin.

**Table A1:** Baseline and postcontrol distributions of risk.

		Risk, control at:		
Decile	Baseline risk	Bottom	Middle	Top
1	3.4 × 10^−4^	3.1 × 10^−4^	3.4 × 10^−4^	3.40 × 10^−4^
2	6.8 × 10^−4^	6.1 × 10^−4^	6.8 × 10^−4^	6.80 × 10^−4^
3	8.0 × 10^−4^	7.2 × 10^−4^	8.0 × 10^−4^	8.00 × 10^−4^
4	8.9 × 10^−4^	8.9 × 10^−4^	8.0 × 10^−4^	8.90 × 10^−4^
5	9.9 × 10^−4^	9.9 × 10^−4^	8.9 × 10^−4^	9.90 × 10^−4^
6	1.1 × 10^−3^	1.1 × 10^−3^	1.0 × 10^−3^	1.10 × 10^−3^
7	1.2 × 10^−3^	1.2 × 10^−3^	1.1 × 10^−3^	1.20 × 10^−3^
8	1.3 × 10^−3^	1.3 × 10^−3^	1.3 × 10^−3^	1.20 × 10^−3^
9	1.4 × 10^−3^	1.4 × 10^−3^	1.4 × 10^−3^	1.30 × 10^−3^
10	1.8 × 10^−3^	1.8 × 10^−3^	1.8 × 10^−3^	1.60 × 10^−3^

Table A2 shows the resulting values of each of the inequality indicators, including the Atkinson index for multiple values of ɛ.

First, it should be noted that the absolute values are less significant than the relative differences between the precontrol and postcontrol scenario. The Atkinson index can take on any value from 0 to 1, depending on the value of ɛ, and the other three inequality indicators represent different conceptualizations of equity.

In all cases, controlling risks at the bottom of the distribution (the lower-risk individuals) led to an increase in the inequality indicators, implying increased inequality of risk. Similarly, in all cases, controlling risks at the top of the distribution (the higher-risk individuals) led to a decrease in the inequality indicators, implying reduced inequality of risk.

Controlling risks in the middle of the distribution was seen as beneficial for some inequality measures and not for others. In particular, inequality increased according to the mean log deviation, the Theil entropy index, Gini coefficient, and Atkinson index for ɛ = 0.5. For higher values of ɛ, inequality according to the Atkinson index decreased. This can be explained by the fact that, for higher values of ɛ, the Atkinson index most heavily penalizes large differences between low values and the mean, and the “control in the middle” scenario has lessened the distance between the mean and the bottom of the distribution, although it has simultaneously increased the distance between the mean and the top of the distribution. As indicated in the text, this emphasizes that the Atkinson index is a somewhat indirect measure for capturing concern about high-risk individuals, as changes in ɛ most directly indicate the degree of concern about low-risk individuals.

**Table A2:** Values of each of the inequality indicators.

		Risk, control at:		
Inequality indicators	Baseline risk	Bottom	Middle	Top
Atkinson, ɛ = 0.5	0.038	0.044	0.040	0.032
Atkinson, ɛ = 1.5	0.127	0.146	0.127	0.112
Atkinson, ɛ = 3	0.282	0.319	0.272	0.259
Atkinson, ɛ = 5	0.445	0.483	0.426	0.423
Mean log deviation	0.084	0.097	0.085	0.072
Theil	0.072	0.083	0.077	0.061
Gini	0.207	0.222	0.215	0.185
